# Influence Mechanisms of Inclusion Types on Rotating Bending Fatigue Properties of SAE52100 Bearing Steel

**DOI:** 10.3390/ma15145037

**Published:** 2022-07-20

**Authors:** Zhiyue Shi, Jingjing Li, Xiaodan Zhang, Chengjia Shang, Wenquan Cao

**Affiliations:** 1 Special Steel Department of Central Iron and Steel Research Institute (CISRI), Beijing 100081, China; b20190517@xs.ustb.edu.cn (Z.S.); lijj17622350716@163.com (J.L.); 2 School of Materials Science and Engineering, University of Science and Technology of Beijing, Beijing 100083, China; cjshang@ustb.edu.cn; 3 Section of Civil and Manufacturing Engineering, Department of Mechanical Engineering, Technical University of Denmark, 2800 Kgs. Lyngby, Denmark

**Keywords:** SAE52100 steel, rotating bending fatigue, inclusion types, stress intensity factor, stess concentration factor

## Abstract

The rotating bending fatigue crack sources of SAE52100 high carbon bearing steel were studied in this paper. On the fatigue fracture surfaces, inclusions for crack initiation are mainly Ca-Al-O and TiN inclusions, and the size of TiN inclusions is much smaller than that of Ca-Al-O inclusions. The analysis of inclusions by Aspex shows that the number and size of Ca-Al-O inclusions in the matrix are much larger than those of TiN inclusions. Combined with the calculation of stress intensity factor and the analysis of stress concentration factor, the sharp angle characteristic of TiN inclusions is the main reason for its stronger deterioration to fatigue properties, and the damaging effect of TiN inclusions with the same size to the matrix fatigue properties is about 1.33–1.67 times that of Ca-Al-O inclusions.

## 1. Introduction

Inclusions have been regarded as one of the key factors affecting the fatigue properties of bearing steels [1]. With the continuous development of smelting technology, the purity of bearing steel has been greatly improved [2]. Previous studies have shown that reducing the average inclusion size from 27 μm to 13 μm through melting processes can improve the rotating bending fatigue strength by about 8.5% and the rolling contact fatigue life by four times [3]. The “equivalent projected area of inclusions” model proposed by Murakami et al. combines the inclusion size with the fatigue crack length to quantitatively analyze the effect of inclusion size on fatigue properties [4]. However, previous studies have focused on the effect of inclusion size on fatigue properties, while there is a lack of quantitative research on the effect of inclusion type on fatigue properties [5]. As we all know, oxide inclusions and nitride inclusions which do not easily deform in bearing steels are the main crack sources leading to fatigue failure [6]. But the physical properties such as shape, elastic modulus and Poisson’s ratio of these two types of inclusions are very different, which will lead to different effects on the fatigue properties of bearing steels [7]. Therefore, it is necessary to quantitatively study the influence of different types of inclusions on the fatigue properties of the same bearing steel in order to guide the accurate control of different types of inclusions in the smelting process of bearing steel.

## 2. Materials and Methods

**Figure 1:**
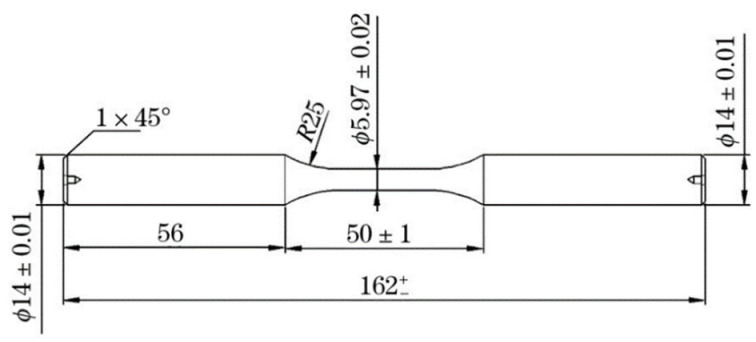
Illustration of sample dimension of the rotated bending fatigue test (the unit is “mm”).

The widely-used high-carbon chromium SAE52100 bearing steel for the machinery manufacturing field [8] was treated in this work by a vacuum induction melting plus electroslag re-melting (VIM + ESR) route with the following chemical composition weight percentage: Fe-1.02C-1.50Cr-0.35Mn-0.25Si-0.0014Ti-0.0053N-0.0012O. All specimens for rotating bending fatigue (RBF) tests and mechanical tests such as tensile tests, impact tests and hardness measurements were austenitized at 840 °C for half an hour, followed by oil quenching, and finally by tempering at 170 °C for 3 h, followed by air cooling. The RBF test was carried out at room temperature in the mechanical fatigue test machine PQ1-6 (Qianbang Test Equipment Co., LTD., Changchun, China) with the stress ratio R = −1 and the resonance frequency of 80 Hz. Both the fatigue strength limit at 1 × 10^7^ cycles and the S–N curve were obtained from the number of cycles and fracture conditions of 30 samples under corresponding loads, and the sample size is shown in Figure 1. Details on fatigue tests, tensile tests and hardness measurements have been reported elsewhere [9,10]. The fracture surfaces were examined by a scanning electron microscope (SEM, S4300, Hitachi, Tokyo, Japan) with an energy dispersive X-ray (EDX) analyzer to investigate the corresponding property-related microstructural features and surface morphology. Inclusion parameters, such as the inclusion type, size and distribution, were quantitatively analyzed by an Aspex Explorer microanalysis system [11]. After heat treatment, the sample was polished and put into Aspex Explorer microanalysis system. The scanning area was set to 120 mm^2^ and the minimum inclusion identification size is 2.5 μm. During the scanning identification process, the size, type, location and composition of each inclusion are determined.

## 3. Results and Discussion

**Figure 2:**
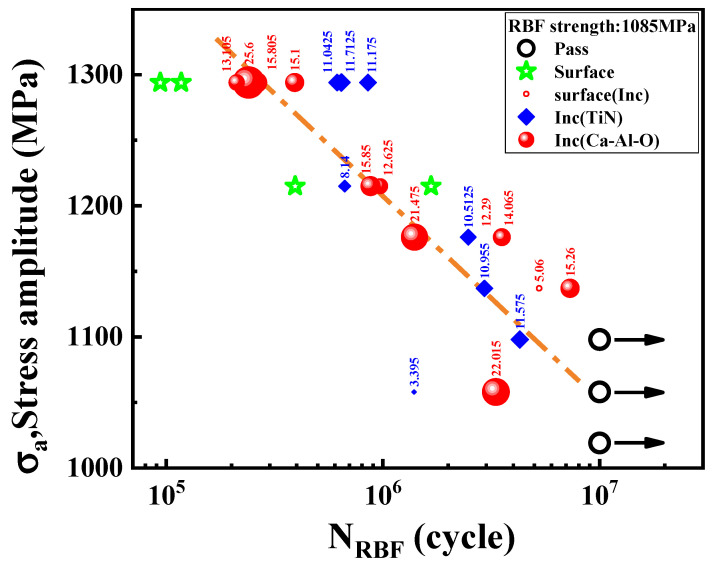
Stress amplitude as a function of rotatory bending cycles for RBF. The numbers are the inclusion sizes with the unit in μm and the size of symbols show the relative inclusion size (see text).

After heat treatment, the tensile strength of SAE52100 bearing steel with VIM + ESR melting routes is 2273 ± 29 MPa, the yield stress is 1775 ± 1.0 MPa, the impact toughness is 10.0 ± 2.0 J and the room temperature hardness is 59.5 ± 1.0 HRC. The stress amplitude (*σ_a_*) as a function of the rotating bending fatigue cycles (*N_RBF_*) of bearing steels are plotted in Figure 2, together with their relative inclusion sizes ($\sqrt{area_{Inc}}$) and types analyzed from the fracture surfaces. It shows that the *N_RBF_* increases with the decrease of *σ_a_*, and the fatigue strength (*σ*_−1_) of bearing steels measured and calculated by the up and down method is 1085 MPa. The inclusions and surfaces of the two groups of samples are the crack sources of fatigue failure. Through the EDS analysis, the inclusion crack sources causing fatigue failure are mainly magnesium aluminates/Al_2_O_3_-CaO-CaS(Ca-Al-O) inclusions and TiN inclusions. The number of Ca-Al-O inclusion crack sources and TiN inclusion crack sources is 11 and 8 respectively, but the average size of nitride inclusion crack source is 9.81 μm, which is much smaller than that of Ca-Al-O inclusion crack source 16.65 μm. The statistics of different types of crack sources under different stress amplitudes levels are shown in Table 1. It can be seen that Ca-Al-O and TiN inclusions will cause fatigue failure under different stress amplitude, while surface failure cracks will only occur under high stress amplitude (1200–1300 MPa). And no matter under high or low stress amplitudes, the crack size of TiN inclusions is smaller than that of Ca-Al-O inclusion, that is, the control factors affecting fatigue crack initiation are not only related to the size of inclusions, but also related to the type of inclusions.

**Figure 3:**
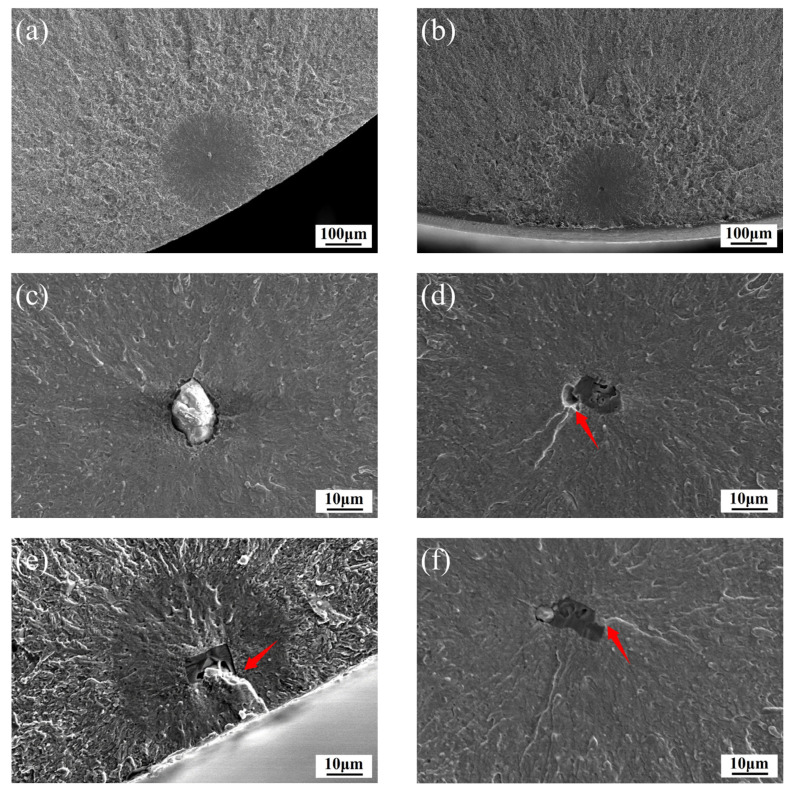
Fracture topography of RBF-failed specimens: (**a**,**c**) the low and high magnification fracture topographies caused by a Ca-Al-O inclusion respectively; (**b**,**d**) the lower and higher magnification fracture topographies caused by a TiN inclusion respectively; (**e**,**f**) TiN inclusion crack source; (**d**,**e**,**f**) the position indicated by the red arrow indicates that cracks preferentially initiate at the sharp corners of TiN inclusions.

The typical inclusions of RBFed fracture samples are demonstrated in Figure 3. Figure 3a,c show the low and high magnification fracture topographies caused by a Ca-Al-O inclusion after 7,307,500 cycles under a stress amplitude of 1137 MPa, Figure 3b,d show the low and high magnification fracture topographies caused by a TiN inclusion after 614,700 cycles under a stress amplitude of 1294 MPa, and Figure 3d–f show the crack source of the TiN inclusion. No matter what type of inclusion crack source, its fracture has obvious fish eye morphology characteristics, that is to say, different types of inclusions mainly affect the crack initiation stage of fatigue processes [12]. The crack source of Ca-Al-O inclusion is spherical, while the TiN inclusion is an irregular polygon with obvious sharp corners [13]. And fatigue cracks tend to propagate from the sharp corners of TiN inclusions, as shown by the red arrow in Figure 3d–f.

**Figure 4:**
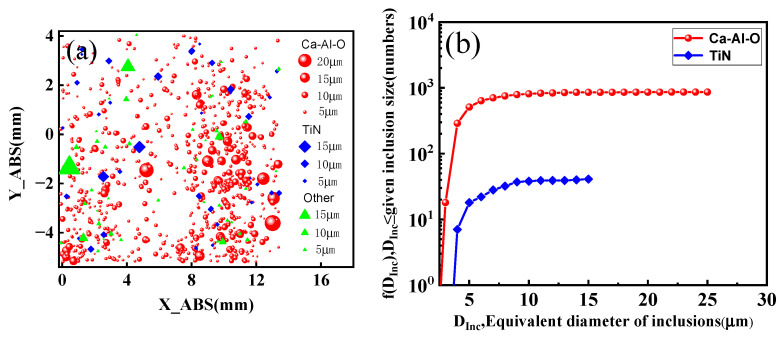
Characterization of inclusions in the bearing steel by an Aspex explorer: (**a**) reconstruction of inclusion size, type and location; (**b**) accumulated size distribution as function of average inclusion.

**Figure 5:**
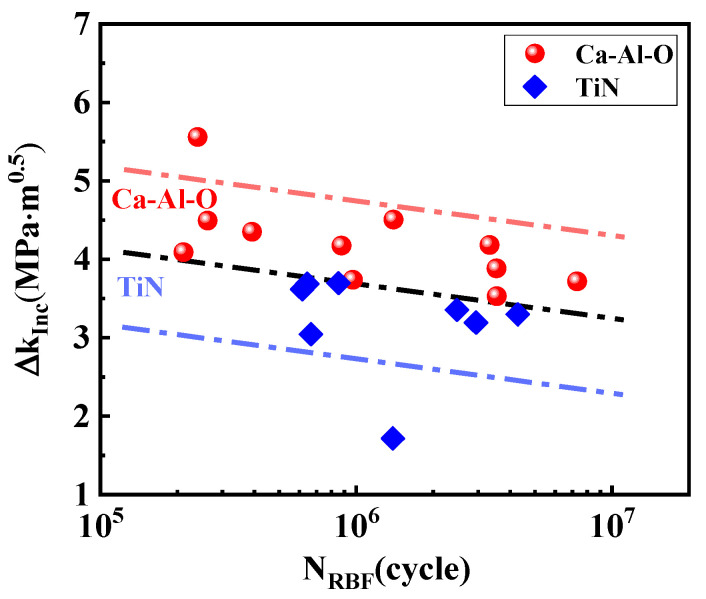
Stress intensity factors of Ca-Al-O and TiN inclusions versus the *N_RBF_*.

**Figure 6:**
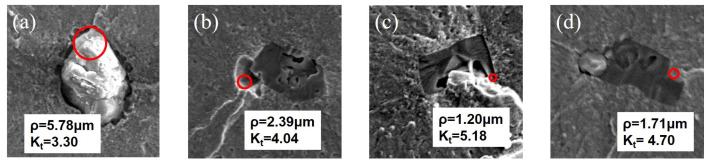
Tip radius of curvature of Ca-Al-O and TiN inclusions: (**a**) Ca-Al-O inclusion and its curvature radius at crack initiation; (**b**–**d**) TiN inclusion and its curvature radius at crack initiation; the circle represents the estimated radius of curvature at the crack initiation.

In order to understand the characteristics of different types of inclusions in the matrix, the inclusion densities are examined by the Aspex Explorer microanalysis system through the reconstruction of inclusion sizes, types and locations, as shown in Figure 4a, and the size of each label represents the relative size of inclusions. The total inclusion number in a measured area of 120 mm^2^ is about 948 for the bearing steel, and the number density of Ca-Al-O and TiN inclusions in bearing steels are 7.16 and 0.34 per square millimeter, respectively. Their corresponding area densities are 8.76 × 10^−3^% and 0.61 × 10^−3^%, respectively. The inclusion size distributions for Ca-Al-O and TiN inclusions in the measured area are shown in Figure 4b, with only the inclusions with an equivalent diameter no less than 2.5 μm in an increment mode. It can be seen that the number of TiN inclusions is 41, and the maximum size is 15.0 μm, while the number of Ca-Al-O inclusions is 861, and the maximum size is 24.1 μm. According to the analysis of Aspex, the TiN inclusion is much smaller than the Ca-Al-O inclusion in terms of number density and area density, but the TiN inclusion can cause the similar RBF failure as the Ca-Al-O inclusion, which shows that the damage of TiN inclusion to the fatigue properties of the matrix is much higher than that of Ca-Al-O inclusion. In order to study the effect of Ca-Al-O and TiN inclusions on fatigue properties, the stress intensity factors (SIF) at inclusions were evaluated using the following equation [5]:

(1) $$\Delta K=F\sigma \sqrt{\pi a}$$

where *F* is the crack shape coefficient (*F* = 0.65 for surface defects and *F* 0.5 for internal defects), *σ* is the stress amplitude, *a* is the crack length (*a =* $\sqrt{area_{Inc}}$). Figure 5 shows the SIF range at Ca-Al-O and TiN inclusions. It can be seen that no matter what type of inclusion, ∆*K_Inc_* decreases with the increase of *N_RBF_* [14]. However, the Ca-Al-O and TiN inclusion are not in the same area, and TiN inclusions are below Ca-Al-O inclusions. In other words, TiN inclusions can cause crack initiation and propagation under smaller SIFs. Ca-Al-O inclusions are mainly spherical, while TiN inclusion are mainly square and have obvious edges and corners. Different geometry will lead to different stress concentrations between inclusions and matrix [15]. The theoretical stress concentration factor *K_t_* calculated by the analytical solution of elastic theory for the large plate sample with elliptical hole is as follows [16]:

(2) $$K_{t}=1+2\sqrt{\frac{A}{\rho }}$$

where *A* is the dimension of long half shaft of elliptical hole (for unified treatment, A=areaInc2), ρ is the tip radius of curvature at the end of the long half axis. The estimated ρ value and $K_{t}$ of two types of inclusion crack sources are shown in Figure 6. For the spherical Ca-Al-O inclusion, its tip radius of curvature is much larger than that of TiN inclusion, and the *K_t_* value is 3.30 as shown in Figure 6a. However, for the TiN inclusion with multi edges and corners, its ρ is much smaller than that of the Ca-Al-O inclusion, so its *K_t_* value is 4.04, 5.18 and 4.70 respectively as shown in Figure 6b–d, that is to say, TiN inclusions have sharper geometric morphology than Ca-Al-O inclusions, so they can cause higher stress concentration [17]. Through the above calculation, the *K_t_* value of the Ca-Al-O inclusion is about 1.41 times that of the TiN inclusion. Therefore, under the same stress amplitude, the crack preferentially initiates at the sharp corner of the TiN inclusion, perpendicular to the direction of tensile stress as shown by the red arrow in Figure 3d–f [18]. After fitting, the intercept of the banded area of the TiN inclusions in Figure 5 is about 0.6–0.75 of that of the Ca-Al-O inclusion, that is, the damage degree of TiN inclusions to the fatigue performance of the matrix is 1.33–1.67 times that of the Ca-Al-O inclusion. This result is basically the same as the *K_t_* value of the two inclusion crack sources, which shows that the stress concentration caused by sharp angle TiN inclusion is the main reason for its small size, but can cause fatigue crack initiation. Table 2 lists the average SIF values of two types of inclusion crack sources under different stress amplitudes. It can be seen that the SIF values of the TiN inclusion is smaller than that of the Ca-Al-O inclusion under different stress amplitudes levels, which indicates that the damage degree of TiN inclusions is higher than that of the Ca-Al-O inclusion under both high stress and low stress conditions. In other words, although TiN inclusions are smaller than Ca-Al-O inclusions in particle size, number density and area density, they will also cause large damages to the fatigue properties of bearing steel due to their sharp angle morphology. The detailed SIF calculation and fracture morphology analysis show that in the high reliability and long-life smelting of bearing steel, not only the size of inclusions should be considered, but also the number of more harmful TiN inclusions should be strictly controlled. How to eliminate the influence of a TiN inclusion on the fatigue properties of bearing steel has become the direction of future research.

## 4. Conclusions

In this work, the fatigue behaviors and mechanical properties of high carbon chromium SAE52100 bearing steel are tested, and the inclusion characteristics are analyzed by SEM and Aspex. Through SEM and Aspex investigations, the inclusions for fatigue failure are mainly Ca-Al-O and TiN inclusions, and the size of the TiN inclusion is much smaller than that of the Ca-Al-O. Combined with the SIF calculation and stress concentration factor analysis, although the size of the TiN inclusion is small, its sharp corner morphology leads to a much larger stress concentration factor higher than that of the Ca-Al-O inclusion. The fatigue damage of TiN inclusions with the same size is about 1.33–1.67 times that of Ca-Al-O inclusions. In the future smelting process of bearing steel, the control of N and Ti content is also one of the key factors affecting the properties of bearing steel.

## Figures and Tables

**Table 1:** Failure modes and inclusion types on fracture surfaces of RBFed specimens examined by SEM.

Stress Amplitude Level (MPa)	Ca-Al-O		TiN		Surface (Number)
	Number	Average Size (μm)	Number	Average Size (μm)	
1000–1100	1	22.02	2	7.49	-
1100–1200	4	15.77	2	10.73	-
1200–1300	6	16.35	4	10.52	4

**Table 2:** Stress intensity factor values of two kinds of inclusion crack sources under different stress amplitude levels.

Stress Amplitude Level (MPa)	Ca-Al-O Average SIF Value	TiN Average SIF Value
1000–1100	4.18	2.51
1100–1200	3.91	3.27
1200–1300	4.40	3.51

## Data Availability

Data sharing is not applicable.
